# O2-3 Feasibility of the Move for Life intervention to help inactive adults 50 years and over increase their physical activity

**DOI:** 10.1093/eurpub/ckac094.011

**Published:** 2022-08-29

**Authors:** Enrique Garcia Bengoechea, Doyle Ciaran, Andrew O'Regan, Liam Glynn, Amanda Clifford, Alan Donnelly, Stephen Gallagher, Nollaig O'Sulllivan, Andrew Murphy, Catherine Woods

**Affiliations:** 1 Physical Education and Sport Sciences, University of Limerick, Limerick, Ireland; 2 Graduate Entry Medical School, University of Limerick, Limerick, Ireland; 3 School of Allied Health, University of Limerick, Limerick, Ireland; 4 Department of Psychology, University of Limerick, Limerick, Ireland; 5 General Practice, National University of Ireland Galway, Galway, Ireland

**Keywords:** community, older adults, implementation, process evaluation, mixed methods

## Abstract

**Background:**

Substantial evidence shows that meeting physical activity guidelines is important for disease prevention, promoting physical and mental health and quality of life. In Ireland, only 38% of older adults are sufficiently active to meet the guidelines. The primary aim of this research was to conduct a feasibility study of the Move for Life intervention to reach and help inactive adults 50+ increase their physical activity.

**Methods:**

Move for Life is a feasibility cluster randomised control trial where the Local Sports Partnerships hubs are the units of randomisation and individuals within the hubs are the units of analysis. The intervention augments regular programme content with behavioural skills, social support and group cohesion strategies, and includes a peer-mentoring component. Over seven hundred (N = 733) participants registered to become part of the intervention. Most (98%, n = 724) completed baseline measures. Participants were asked to complete self-report process evaluation questionnaires immediately after and 12 weeks after their programme had finished. Interviews were conducted with participants, physical activity instructors and peer mentors. Questionnaire responses across groups were compared with tests of statistical significance. Qualitative data complemented and assisted with interpretation of quantitative findings.

**Results:**

A total of 601 participants met eligibility criteria (average age 63.06 years, range 50-91, 80.4% female). The study retention rate was 63%. Reported compliance rate with intervention strategies was over 75%. The intervention group were more likely to enjoy the programme than usual provision. Intervention participants were more likely to report the programme as interesting and worth the time they invested in it, and to recommend it to a friend than usual provision. They were also more likely to report enjoying learning about different strategies to keep physically active (ORs ranging from 2.56 to 3.76). At 3-month follow up, the intervention group were more likely to be aware of their Local Sports Partnership opportunities and to have contacted their Local Sports Partnership more frequently in comparison to usual provision (all p >.05).

**Conclusions:**

Move for Life is a realistic and promising augmentation to existing physical activity programmes for older adults with potential for adoption and scalability following completion of a full trial.

